# Patients treated with thinner artificial hair implants: a case series

**DOI:** 10.3389/fmed.2025.1717658

**Published:** 2025-12-03

**Authors:** Antonio Scarano, Maria Vadalà, Sergio Alexandre Gehrke, Beniamino Palmieri

**Affiliations:** 1 Department of Innovative Technologies in Medicine & Dentistry, University of Chieti-Pescara, Chieti, Italy; 2 Second Opinion Medical Network, Modena, Italy; 3 Medico Cura Te Stesso Onlus, Murcia, Spain; 4 Department of Biotechnology, Universidad Católica de Murcia (UCAM), Murcia, Spain

**Keywords:** hair implant, hair surgery, alopecia, FUT, PRP, LED, biofiber, baldness

## Abstract

**Background:**

Artificial biocompatible hair implants have been developed and clinically evaluated as a solution for hair loss, utilizing various materials and techniques to optimize safety, efficacy, and patient satisfaction. The primary objective of this study was to assess patient satisfaction in individuals affected by alopecia and treated with Biofibre^®^ 4.0, focusing on scalp hair density and aesthetic perception using a four-level evaluation scale. The secondary objective was to evaluate the rate of implanted hair loss at the one-year follow-up.

**Materials and methods:**

A total of 15 patients from different ethnicities were enrolled in the study, including 10 male and 5 female individuals. Biocompatible artificial hair with a diameter of 0.08 mm and a length between 160 and 460 mm (Biofibre^®^ 4.0, Medicap^®^, Carpi, Italy) were utilized in the present study. The root of each fiber was positioned beneath the scalp, specifically at the level of the galea aponeurotica.

**Results:**

Biocompatibility was excellent, with no reported allergies or significant “foreign body” skin reactions. Analysis based on the primary effectiveness endpoint revealed a mean score of 3.1 on a 4-point grading scale, where 1 = not satisfied (one patient), 2 = somewhat satisfied (two patients), 3 = satisfied (seven patients), and 4 = very satisfied (five patients).

**Discussion:**

The implantation of new, thinner artificial hair fibers represents a valid and safe technique. Tolerability has improved due to the increased softness and elasticity of these fibers.

**Conclusion:**

In conclusion, although the sample size is small, the results of this case series confirm the efficacy and safety of the new Biofibre^®^ 4.0 in the treatment of both male and female androgenetic alopecia (AGA), offering a well-tolerated and effective solution for improving aesthetic appearance and psychological well-being.

## 1 Introduction

Alopecia refers to a group of disorders characterized by hair loss, which can affect the scalp or other parts of the body and may be localized or diffuse, and it can be permanent or temporary. The causes of alopecia are highly diverse and include genetic predisposition, hormonal imbalances, autoimmune mechanisms, nutritional deficiencies, stress, infections, certain medications (such as chemotherapy), and physical or chemical trauma to the hair or scalp (1). Alopecia has an estimated prevalence of 1.7%–2.1% in the general population and tends to appear predominantly after puberty, with a gradual progression over time (2). Patients may experience significant distress due to facial aesthetic changes, which can negatively affect their quality of life (3).

Alopecia, regardless of its etiology, often leads to psychological distress, including potential loss of self-esteem due to changes in facial appearance. The social context may play an important role in shaping an individual’s ability to tolerate and accept this condition (4). A wide range of therapies is available for the treatment of alopecia, with approaches tailored to the specific type and severity of the condition. These therapies include topical and systemic treatments (5), light and laser therapies (6), and systemic and topical immunotherapy (7). Surgical techniques for hair restoration include several options, such as follicular unit transplantation, scalp reduction, hair transplant, and artificial biocompatible hair implants (8, 9). The most common surgical treatment for alopecia is hair transplantation [follicular unit transplantation (FUT)/follicular unit extraction (FUE)]. FUT involves removing a strip of scalp to extract follicular units and is suitable for larger hair transplant sessions. FUE involves individually extracting follicular units directly from the donor area and is preferred for minimal scarring and faster recovery. Both techniques are widely used in hair transplant surgery (10). A gentle alternative for hair restoration is provided by biocompatible hair implants (11–13). Artificial biocompatible hair implants have been developed and clinically evaluated as a solution for hair loss, utilizing various materials and techniques to optimize safety, efficacy, and patient satisfaction. Biofibre and other artificial hair fibers are modern, medical-grade fibers developed for hair restoration, particularly in cases where traditional transplantation is not feasible (14).

Recently, a new fiber has been proposed that is slightly thinner and exhibits reduced capillarity, which helps lower the risk of bacterial adhesion and, consequently, infection.

The primary objective of this study was to assess patient satisfaction in individuals affected by alopecia and treated with Biofibre^®^ 4.0, focusing on scalp hair density and aesthetic perception using a four-level evaluation scale. The secondary objective was to evaluate the rate of implanted hair loss at the one-year follow-up.

## 2 Presentation of the cases

A total of 15 patients from different ethnicities were enrolled in the study, including 10 male and five female individuals. The inclusion criteria were as follows: Age 25–70 years, both male and female individuals, a dermatologically confirmed diagnosis of androgenetic alopecia (AGA), and grading according to the Hamilton scale. The exclusion criteria included the following: Individuals with psychological disorders, dermatitis or dermatosis of the scalp, metabolic disorders, immunodeficiencies, and allergies; patients not willing to return for follow-up or with reduced therapeutic compliance; and cases in which proper hygiene could not be guaranteed or maintained.

All patients were in good general health, with particular attention given to scalp physiology. They were highly motivated to pursue artificial hair replacement and were available for regular follow-up visits for check-up and proper medication management. Informed consent was obtained from all patients. Biocompatible artificial hair with a diameter of 0.08 mm and a length between 160 and 460 mm (Biofibre^®^ 4.0, Medicap^®^, Carpi, Italy) were utilized in the present study. The implantation procedure is a surgical technique performed using either a manual implanter or an automatic device under local anesthesia. The root of each fiber is positioned beneath the scalp at the level of the galea aponeurotica. At the root of the fibers, there is a reversible knot that ensures secure anchorage of the implant and allows for complete removal of the fiber if necessary.

The first step for each patient involved a clinical evaluation and diagnosis, which included medical history, psychological assessment, dermatoscopic examination, and routine blood tests. In total, 10 patients were implanted with fibers measuring 15 cm in length, and 5 patients received fibers measuring 30 cm in length.

Biocompatible hair is implanted under superficial local anesthesia using a special instrument called automatic implanter, which allows the hair to be implanted at the aponeurotic level. This device allows better cicatrization and easier practice (5, 6), according to the modern medical protocol of this technique (7, 8). Biocompatible hair implantation begins with a compatibility test of 100 hairs. After 5 weeks, if no reaction is observed around the implanted fibers, additional implant sessions are performed, each comprising 500 fibers spaced 2 mm apart in a reticular pattern.

The color of the fiber must match the patient’s hair colour. The orientation of the fiber must respect the natural orientation of the patient’s hair. The total amount of fibers successfully implanted good, and patient satisfaction with hair replacement was individually planned and tailored. After the implant, topical antibiotic therapy was prescribed for 10 days (ciprofloxacin 500 mg twice a day), along with shampoo containing chlorhexidine and hyaluronic acid. For proper scalp care, we recommended that the patients use lukewarm water and dry hair naturally; avoid scratching the scalp; and, starting 15 days after the implant, use a neutral shampoo daily and a ketoconazole shampoo once a week (or a similar alternative), along with gentle periodic tri-weekly skin friction using a soft toothbrush. Local clindamycin lotion was prescribed for daily use, or every 2 days in cases of perspiration or itching. A form containing the post-operative guideline, as well as a list of prohibited treatments, habits, and products, was provided to each patient after the surgical procedure.

The interval between sessions averaged 5 weeks across the different scalp areas, each preceded by a check-up of previous outcomes and by regular compliance with after-care instructions. A good aesthetic result is usually achieved after 2–3 sessions, with a total of 1,500 hairs implanted per patient. Implantation in areas with frontal muscle, as well as in the temples and temporal areas, should be avoided. Customized, careful patient post-operative care is needed for optimal results. Follow-up clinical sessions start 48 h after the first procedure, then at 1 month, and subsequently every 3 months for 1 year after surgery. To assess the loss of implanted hair, 1 cm^3^ areas were selected. The severity of baldness was classified using subjective assessment tools, specifically the Sinclair scale for the female patients and the Hamilton–Norwood scale for the male patients.

### 2.1 Statistical analysis

The data were recorded using a specially designed electronic database (Excel, Microsoft 360, Redmond, WA, USA). Data analysis was performed using the software package GraphPad 9 (Prism, San Diego, CA, USA). Descriptive statistics were calculated, including means, medians, interquartile ranges, and 95% confidence intervals, for all treatment groups. The interactions between the dependent variable (grade of baldness) and independent variables (e.g., age, job, skin phenotype, and ethnicity) were analyzed using multiple linear regression. The Wilcoxon test was used to assess the level of significance before and after treatment, with a *p*-value of <0.05 considered statistically significant.

## 3 Results

Male subjects had a mean baldness grade of 4 ± 0.82 according to the Hamilton-Norwood scale, while female subjects had a mean baldness grade of 3.2 ± 0.84 according to the Sinclair scale.

### 3.1 Population characteristics

A total of 15 participants were enrolled in the present study, including 10 male (66.7%) and five female individuals (33.3%), with a mean age of 50.4 ± 13.8 years (Median: 50 years; Range: 29–69 years; 95%CI: 42.7–58.1 years). The participants were categorized according to their job, including eight employees or not working (53.3%), five with a graduate-level education (33.3%), and two workers (13.3%). According to ethnicity, three participants (13.3%) were Asian and 12 were Caucasian (86.7%). Skin phototypes, according to the Fitzpatrick scale, showed a mean of 3.27 ± 1.1 (Median: 3; Range: 2–5; 95%CI: 2.66–3.88).

### 3.2 Treatment findings

#### 3.2.1 Male group

The mean age of the sample was 45.7 ± 13.9 years (Min-Max: 29–66 years; 95%CI: 35.8–55.6) (*p* = 0.0297). According to ethnicity, eight were Caucasian and two were Asian (*p* = 0.0391). No significant associations were observed between the grade of baldness and the independent variables of occupation (*p* = 0.4572) or skin phenotype (*p* = 0.5263).

According to the Hamilton–Norwood scale, the grade of baldness in the male group before treatment was 4 ± 0.82 (median: 4; 95%CI: 3.4–4.6; ir: 3–5). After treatment, the mean grade reduced to 2.6 ± 0.52 (median: 3; 95%CI: 2.2–3; ir: 2–3) (*p* = 0.0020).

**Figure 1:**
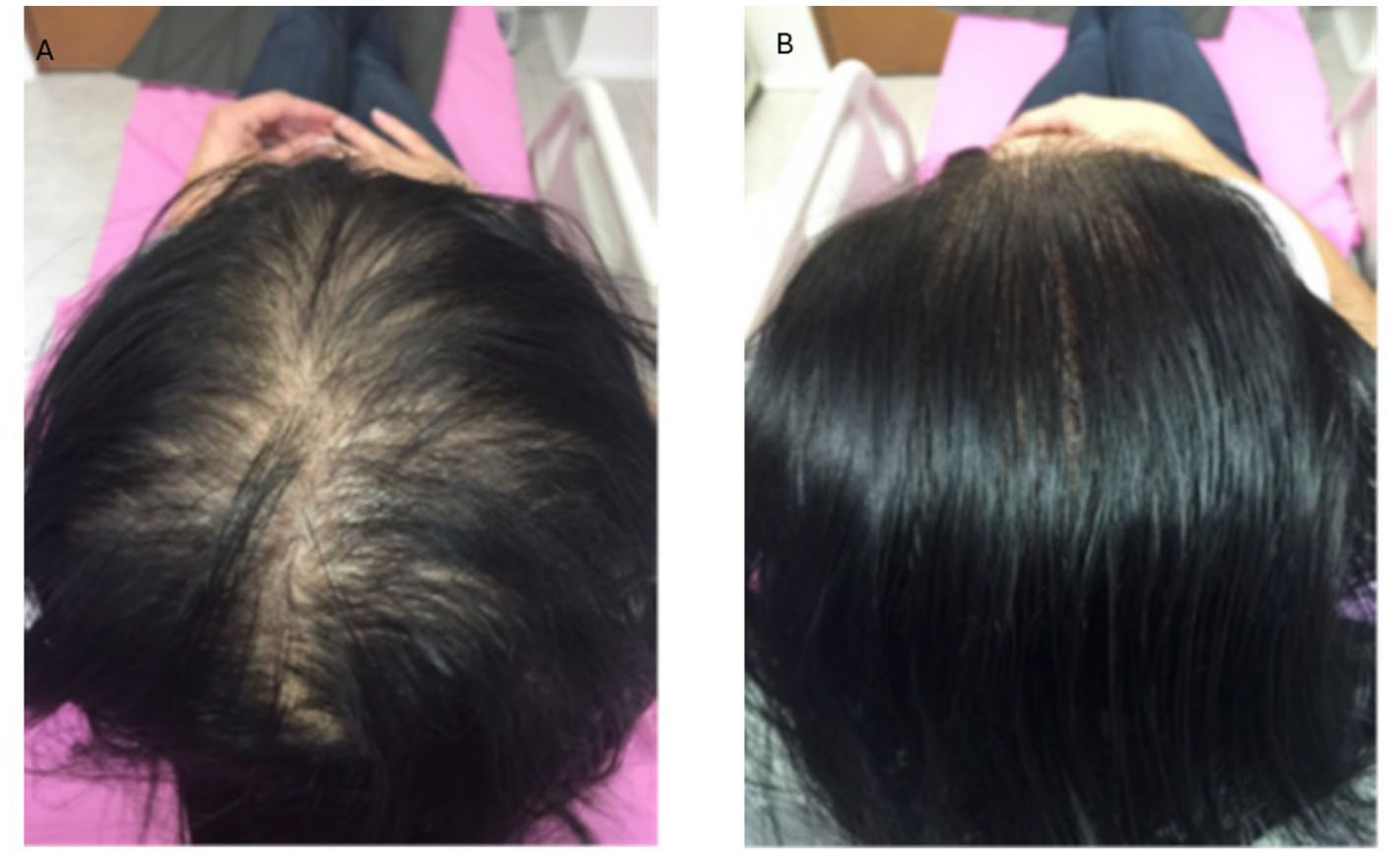
Grade 2 hair loss according to the Sinclair scale. The female patients before **(A)** and after **(B)** biocompatible hair implantation.

**Figure 2:**
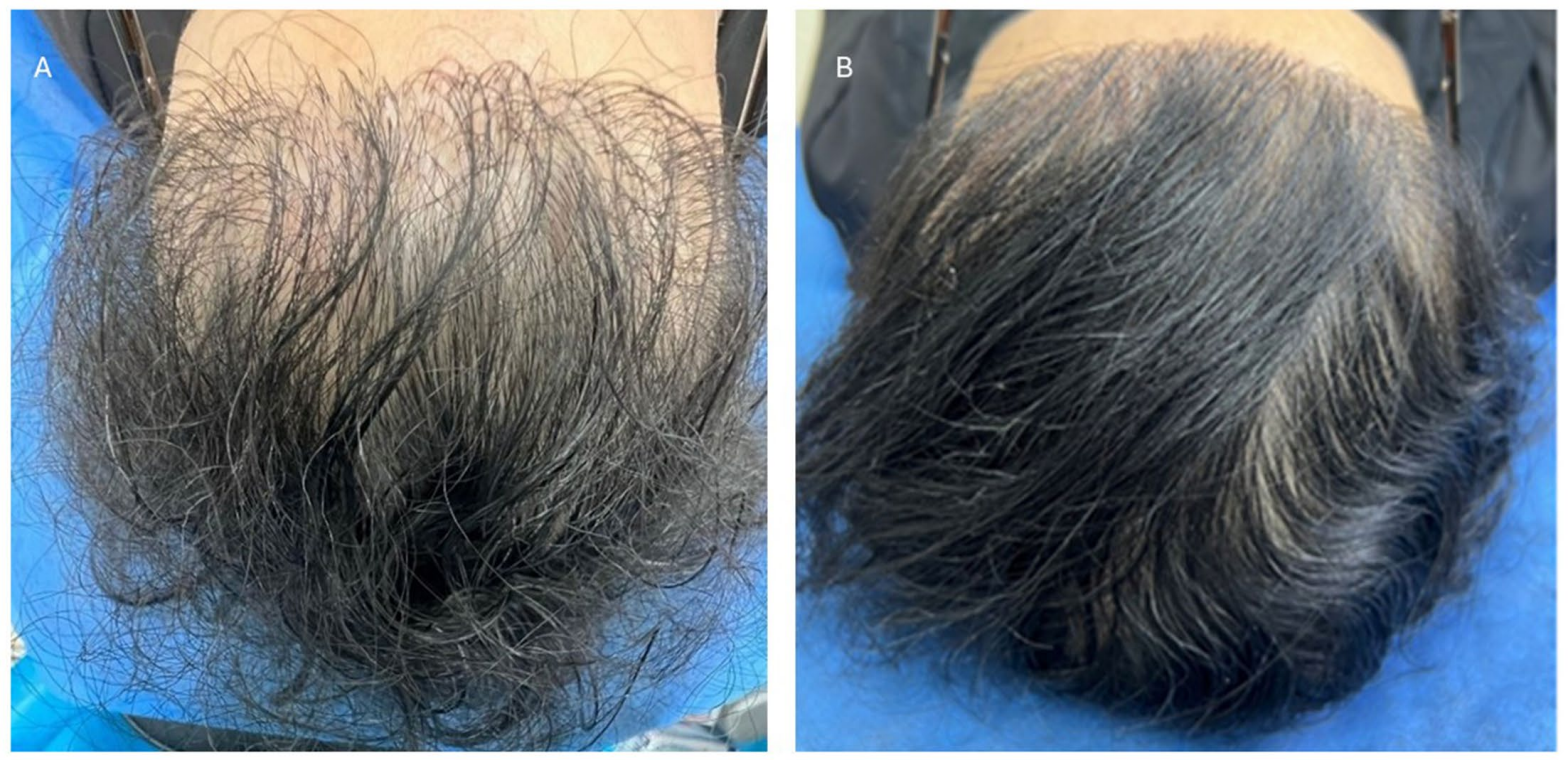
Grade 2 hair loss according to the Sinclair scale. The female patients before **(A)** and after **(B)** biocompatible hair implantation.

**Figure 3:**
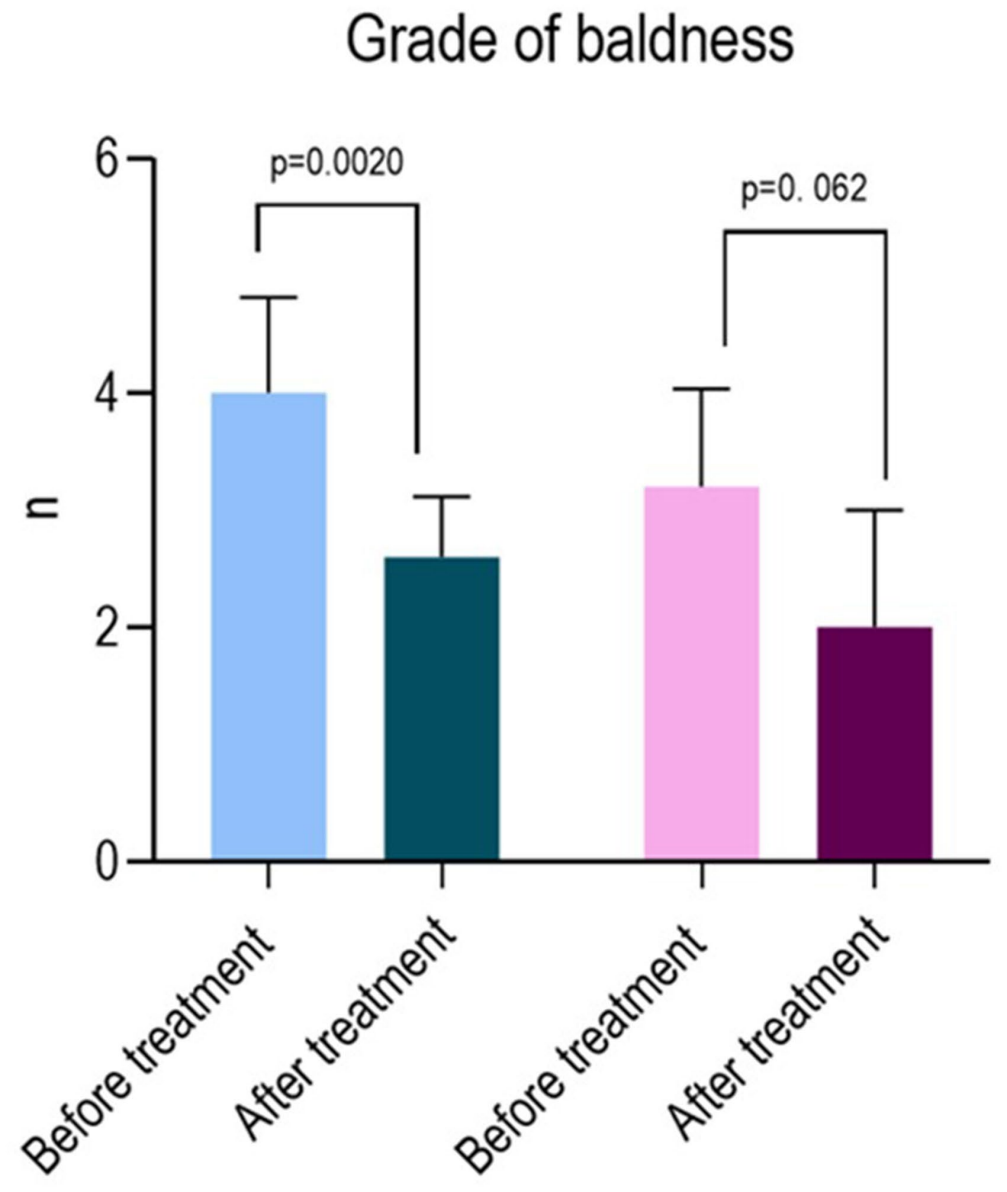
Chart summary of the level of significance tested before and after treatment. The population was stratified by sex, considering separately the male and female populations.

The implantation procedure with the new fibers was straightforward, completely safe, and uneventful for our group of patients (Figures 1–3). Biocompatibility was excellent, with no allergies or significant “foreign body” skin reactions. The clinical data and follow-up outcomes are summarized in Tables 1–4.

**Table 1:** Patient distribution by sex, age, occupation, grade of baldness (Hamilton–Norwood Scale), skin phototype (Fitzpatrick scale), and ethnicity.

Pt	Gender	Age	Job	Grade of baldness Hamilton/Norwood scale	After biocompatible hair implantation	Grade of baldness Sinclair scale	After biocompatible hair implantation	Phenotype Fitzpatrick scale	Ethnicity*
#1	M	30	G	4	3			3	CA
#2	M	42	G	4	3			2	AS
#3	M	33	W	5	3			5	CA
#4	M	63	E	3	2			3	CA
#5	M	50	E	4	3			5	AS
#6	M	61	G	3	2			2	CA
#7	M	66	E	3	2			5	CA
#8	M	46	E	4	2			3	CA
#9	M	29	W	5	3			4	CA
#10	M	37	G	5	3			2	CA
#11	F	55	G			4	3	3	CA
#12	F	59	E			3	2	3	AS
#13	F	49	E			4	3	2	CA
#14	F	67	E			2	1	4	CA
#15	F	69	E			3	1	3	CA

E, employee/not working; G, graduate; CA, Caucasian; AS, Asian; W, worker. *The symbol indicates the worker.

**Table 2:** Schematic representation of clinical characteristics, complications, timing, and healing time of the patients enrolled in the study.

Pt	Average hair for session	Total qty	Complications	Timing	Therapy	Healing time
#1	433	1,300	//	//	//	//
#2	400	1,200	//	//	//	//
#3	475	1,900	Mild infection	At 4 months	Local antibiotic	8 Days
#4	400	800	//	//	//	//
#5	550	1,100	//	//	//	//
#6	500	1,000	//	//	//	//
#7	450	900	//	//	//	//
#8	466	1,400	//	//	//	//
#9	566	1,700	//	//	//	//
#10	450	1,800	Mild inflammation	At 6 months	Local steroid	10 Days
#11	550	2,200	//	//	//	//
#12	433	1,300	//	//	//	//
#13	575	2,300	Mild inflammation	At 7 months	Local steroid	6 Days
#14	400	400	//	//	//	//
#15	450	1,800	//	//	//	//

**Table 3:** Summary of the grade of baldness measured before and after treatment for both sex groups, analyzed using the Wilcoxon test.

	Male group		Female group	
	Hamilton/Norwood scale		Sinclair scale	
	Before treatment	After treatment	Before treatment	After treatment
Mean	4	2.6	3.2	2
Std. deviation	0.82	0.52	0.84	1
Std. error of mean	0.26	0.16	0.37	0.45
25% Percentile	3	2	2.5	1
Median	4	3	3	2
75% Percentile	5	3	4	3
Lower 95% CI of mean	3.4	2.2	2.2	0.76
Upper 95% CI of mean	4.6	3	4.2	3.2
*p* value Wilcoxon test	*p* = 0.0020		*p* = 0.062	

**Table 4:** Summary of the multiple linear regression analysis for the male participants.

M. Linear regression Male group Hamilton/Norwood scale		
		*p* value
A: Intercept	-	*0.0007
B: Age	Mean: 45.7 ± 13.9 yo (Min-Max: 29–66 yo; 95%CI: 35.8–55.6)	*0.0297
C: Job	E: 40%; G: 40%; W: 20%	0.4572
D: Phenotype Fitzpatrick scale	Mean: 3.4 ± 1.26; Median 3.0 (Min-Max: 2–5; 95%CI: 2.5–4.3)	0.5263
E: Ethnicity*	CA:80%; AS:20%	0.0391

The dependent variable (grade of baldness) was analyzed in relation to the study’s independent variables: age, sex, job, skin phenotype, and ethnicity. *The symbol indicates statistical significance.

After implantation, all cases showed acute inflammation in the form of infiltrates of low density localized around the implanted fibers. Over time, these infiltrates changed into connective tissue, which grew around the knot, securing the hair in place. The annual hair loss was approximately 10% for 15 cm fibers and 15% for 30 cm fibers. Mild inflammation was observed only in three patients who received between 1,800 and 2,300 Biofibre implants, whereas no signs of inflammation were noted in the patients who underwent implantation of fewer than 1800 Biofibre units.

#### 3.2.2 Female group

The mean age of the sample was 60 ± 8.3 years (Min-Max: 39–69 years; 95%CI: 49–70) (NA). According to ethnicity, four were Caucasian and one was Asian. In the subgroup, one participant had a graduate-level education and four were employees or not working. No significant associations were observed between the grade of baldness and all independent variables.

According to the Sinclair scale, the grade of baldness reported in the female group before treatment was 3.2 ± 0.84 (Median: 3; 95%CI: 2.2–4.2; ir: 2.5–4). After treatment, the mean grade reduced to 2 ± 1 (Median: 2; 95%CI: 0.76–3.2; ir: 1–3) (*p* = 0.062) (Table 5 and Figure 3).

**Table 5:** Summary of the multiple linear regression analysis for the female participants.

M. Linear regression Female group Sinclair scale		
		*p* value
A: Intercept	–	–
B: Age	Mean: 60 ± 8.3 (Min-Max:49–69; 95%CI: 49–70)	–
C: Job	E: 80%; G: 20%	–
D: Phenotype Fitzpatrick Scale	Mean: 3 ± 0.71; Median 3 (Min-Max: 2–4; 95%CI: 2.1–3.9)	–
E: Ethnicity	CA:80%; AS:20%	–

The dependent variable (grade of baldness) was analyzed in relation to the study’s independent variables: age, sex, job, skin phenotype, and ethnicity.

### 3.3 Degree of patient satisfaction

We used a scoring system to assess the degree of aesthetic improvement compared to the baseline condition. The scale included the following categories:

4 = very satisfied, 3 = satisfied, 2 = quite satisfied, 1 = no change, and 0 = not satisfied.

Analysis based on the primary effectiveness endpoint revealed a mean score of 3.1 on a 4-point grading scale, where 1 = not satisfied (one patient), 2 = somewhat satisfied (two patients), 3 = satisfied (seven patients), and 4 = very satisfied (five patients).

All treated patients experienced a noticeable improvement in hair density, a result that was widely appreciated. A significant increase in scalp coverage was observed in previously thinning areas. This effect contributed to enhancing patients’ self-perception, reducing the emotional impact associated with hair loss, and increasing overall satisfaction with the treatment (Figures 1–3).

## 4 Discussion

The safety endpoint of our investigation was the total adverse event rate (“any complication”) over a one-year follow-up period. The primary effectiveness endpoint was patient satisfaction in terms of scalp density restoration and aesthetic perception, assessed using a 4-point grading scale. Only mild inflammation was observed in three patients who received between 1,800 and 2,300 fiber units, and it resolved within a few days with local treatment. This finding may suggest that implanting a high number of artificial hairs in a single session could increase the risk of inflammatory reactions; therefore, the quantity of fibers should be carefully considered during the planning phase. The secondary effectiveness endpoint was to assess the annual average rate of fiber loss. When the results were analyzed according to the primary effectiveness endpoint, an average score of 3.1 was obtained on a 4-point grading scale, where 1 = not satisfied (one patient), 2 = quite satisfied (two patients), 3 = satisfied (seven patients), and 4 = very satisfied (five patients). All patients, both male and female, showed an improvement of at least one grade on the baldness classification scale used. The satisfaction level expressed by the patients refers to the patients’ expectations. Regarding the secondary effectiveness endpoint, the annual hair loss was approximately 10% for 15 cm fibers and 15% for 30 cm fibers. This result is in line with expectations and with the findings reported by other authors (13). A recent study showed that the average annual loss of implanted fibers is approximately 10%, indicating good long-term retention and biocompatibility (15). Careful post-operative scalp hygiene, along with the use of appropriate products, plays a crucial role in minimizing hair shedding and preventing recurrent folliculitis (16). The technique demonstrates a favorable safety profile, with over 90% of cases free from significant complications. The biocompatible artificial hair with a diameter of 0.08 mm used in the present study are slightly thinner than those previously used, which makes them softer without resulting in an increased rate of complications.

Biofibre^®^ 4.0 artificial hair is a CE MDR-approved medical device with special features. It is considered safe, well-tolerated by the human body, and completely extractable if necessary. Its aesthetic characteristics are very similar to natural hair. It is characterized by three lengths, three different shapes (straight, wave, and curly), and multiple colors that can be customized to meet patient preferences. Its mechanical resistance and elasticity ensure a good duration. It is also available in a high-density variation to improve volume in the crown area. A 12-month follow-up after the last implant session was established based on our extensive surgical experience to evaluate annual hair loss and possible reactions. After implanting artificial hair, the following complications may occur: infection, sebum gland hypersecretion, local inflammatory reactions at the implanted areas, and curling. The above-mentioned complications can be of different degrees according to patients’ individual habits and responses (9, 10). With this new generation of fibers, we were able to demonstrate that the formation of comedones was almost completely prevented. This is probably due to the smaller diameter of the fibers, along with greater elasticity, which allows the pseudo-infundibulum to seal more tightly, thereby reducing sebaceous reactions and sebum storage. The cosmetic outcome of these fibers, although thinner, is not inferior to that of the previous, larger fibers. However, the volumizing effect on the implanted scalp is somewhat reduced, since the softer fibers lie closer to the scalp, similar to natural hair. This does not appear to have any negative psychological impact on the patients. On the other hand, the tensile strength is consistent with that of the previous fibers, allowing long-term implant stability and mechanical resilience. According to the safety endpoint, we observed that inflammatory reactions were rare and mild: topical steroid therapy was required for 1 week in only two cases. No significant infectious complications were detected. One patient experienced a mild side effect, presenting with small vesicular eruptions, which resolved after 1 week of local antibiotic therapy. No patients required implant removal. Several clinical studies have demonstrated the efficacy and safety of Biofibre^®^ artificial hair implantation in patients affected by alopecia. In a large-scale study involving 1,518 patients, the treatment yielded immediate and visibly appreciable results, with a notably high rate of aesthetic satisfaction and improved psychological well-being, underscoring its positive impact on patients’ quality of life (15). Further supporting these findings, a prospective study involving 213 patients reported that 97.94% of participants experienced a significant improvement in scalp coverage and self-perception. Importantly, only 9.27% of patients reported side effects, all of which were described as mild and manageable, confirming the procedure’s favorable safety profile (16).

In addition, another clinical investigation reported an 85% success rate in restoring the appearance of hair, with a low average failure rate of less than 20%, further validating the consistency and reliability of the Biofibre^®^ implantation technique (17). Clinical evidence supports the safety and efficacy of Biofibre^®^ artificial hair implantation as a reliable solution for patients experiencing androgenetic alopecia. In particular, a high success rate has been documented, with the vast majority of patients reporting not only significant improvements in hair density and coverage but also a marked enhancement in psychological well-being and self-esteem. Moreover, it is associated with high levels of patient satisfaction and notable psychological benefits, making it a reliable and well-tolerated option for hair restoration.

Compared to previous generations of fibers, improved skin tolerance and easier post-implant management with less peri-implant hyperkeratosis/parakeratosis were observed. The reduced units implant session and the softness of the implanted fibers enabled the prevented use of antibiotic therapy. Monthly hair loss was in line with that observed with previous fibers (18). The formation of comedones was reduced by approximately 40%, both in terms of quantity, size, and persistence compared to previous studies (19). The removal of these comedones is much easier and eliminates the risk of compromising the fiber. In fact, sebum surrounds the extremely small implant hole with a tiny layer, without pooling and with minimal adherence to the artificial hair units. We are strong advocates of artificial hair implantation in appropriately selected cases, ensuring rigorous patient selection and commitment to proper hair care during the postoperative period, throughout follow-up, and in the long term. Patients with inflammatory or degenerative skin diseases, a history of diabetes or allergies, or immunological impairments are generally not suitable for this procedure. Our soft, microinvasive procedure does not result in scar formation, can be applied to atrophic areas, including scars (20, 21), and can be combined with other treatments, improving patient self-confidence (22). It can restore hair density even after follicular autotransplantation, when the result obtained is not satisfactory. As an ancillary procedure of hair autotransplantation, the proper distance between natural and artificial hair must be maintained to avoid bulb ischemia. Before performing the artificial hair implant procedure, full healing of the transplanted bulbs and the emergence of new hair from them must be achieved. The best solution for alopecia often involves a combination of multiple therapies and treatments to ensure that each patient achieves a customized and most effective outcome. Mesotherapy, for example, can be administered preoperatively to enhance dermal thickness, providing a more stable anchor for the fiber knot. Soft laser and LED devices are also beneficial, both pre- and postoperatively, as they biologically stimulate the skin’s reparative potential and help balance the epithelial layer (23). The outcomes of the present study highlight the dual benefits of the procedure: the restoration of a natural-looking hair appearance and the positive impact on patients’ emotional and social lives. Biofibre hair implants have demonstrated a low incidence of long-term complications, with only 8.75% of patients experiencing minor adverse effects, such as localized inflammation. No permanent damage or scarring was observed during the follow-up period, confirming an overall favorable safety profile (22).

Mild infections occurred in 5.9% of cases, while inflammatory reactions were reported in 3.8%, mainly attributed to the incorrect use of chemical substances. Complications resolved in 97.9% of cases within an average of 15 days and were typically managed with antibiotics or corticosteroids (13). When necessary, fiber removal was feasible without residual scarring (19).

These findings contrast with data from earlier literature, which reported significantly higher complication rates and frequent permanent damage, including fiber rejection, chronic infections, facial edema, persistent pain, pruritus, deep scarring, and irreversible loss of natural hair (24). Moreover, long-term risks, such as potential carcinogenesis due to deeply embedded synthetic fibers, were also highlighted (24). Such differences underscore the technological advancements in both implantation techniques and materials, leading to a marked reduction in adverse events and an improved safety profile in contemporary clinical practice (18, 22, 25). The minimally invasive nature of the technique, combined with its low incidence of adverse effects, further reinforces its profile as a safe and effective therapeutic option for individuals seeking aesthetic and psychological improvement. Several studies have shown that this innovative technique provides immediate and visible results, coupled with high levels of patient satisfaction and a low incidence of complications. These characteristics make Biofibre^®^ implantation a viable and attractive option for individuals seeking a non-surgical hair restoration solution, particularly for those who are not ideal candidates for traditional hair transplantation due to insufficient donor hair availability. Despite the one-year follow-up, the present study is limited by its small sample size, consisting of 10 male and five female individuals.

In conclusion, despite the small sample size, the results of this case series confirm the efficacy and safety of the new Biofibre^®^ 4.0 in the treatment of both male and female androgenetic alopecia, offering a well-tolerated and effective solution for improving aesthetic appearance and psychological well-being.

## Data Availability

The original contributions presented in the study are included in the article/supplementary material, further inquiries can be directed to the corresponding author.
